# Bioassay-guided discovery of antibacterial agents: in vitro screening of Peperomia vulcanica, Peperomia fernandopoioana and Scleria striatinux

**DOI:** 10.1186/1476-0711-11-10

**Published:** 2012-05-01

**Authors:** James A Mbah, Moses N Ngemenya, Ashime Louis Abawah, Smith B Babiaka, Lina N Nubed, Kennedy D Nyongbela, Njimoh Dieudonne Lemuh, Simon MN Efange

**Affiliations:** 1 Department of Chemistry, Faculty of Science, University of Buea, P.O. Box 63, Buea, Southwest Region, Cameroon; 2 Department of Biochemistry and Microbiology, Faculty of Science, University of Buea, P.O. Box 63, Buea, Southwest Region, Cameroon

**Keywords:** Resistance, Medicinal plants, Antibacterial compound, Toxicity

## Abstract

**Background:**

The global burden of bacterial infections is high and has been further aggravated by increasing resistance to antibiotics. In the search for novel antibacterials, three medicinal plants: Peperomia vulcanica, Peperomia fernandopoioana (Piperaceae) and Scleria striatinux (Cyperaceae), were investigated for antibacterial activity and toxicity.

**Methods:**

Crude extracts of these plants were tested by the disc diffusion method against six bacterial test organisms followed by bio-assay guided fractionation, isolation and testing of pure compounds. The minimum inhibitory (MIC) and minimum bactericidal (MBC) concentrations were measured by the microdilution method. The acute toxicity of the active extracts and cytotoxicity of the active compound were performed in mice and mammalian cells, respectively.

**Results:**

The diameter of the zones of inhibition (DZI) of the extracts ranged from 7–13 mm on Escherichia coli and Staphylococcus aureus of which the methylene chloride:methanol [1:1] extract of Scleria striatinux recorded the highest activity (DZI = 13 mm). Twenty-nine pure compounds were screened and one, Okundoperoxide, isolated from S. striatinux, recorded a DZI ranging from 10–19 mm on S. aureus. The MICs and MBCs indicated that the Peperomias had broad-spectrum bacteriostatic activity. Toxicity tests showed that Okundoperoxide may have a low risk of toxicity with an LC_50_ of 46.88 μg/mL.

**Conclusions:**

The antibacterial activity of these plants supports their use in traditional medicine. The pure compound, Okundoperoxide, may yield new antibacterial lead compounds following medicinal chemistry exploration.

## Introduction

Bacterial infections account for a significant proportion of the global infectious disease burden, along with a high morbidity and mortality [1], and impact negatively on human welfare and the economy. Antibiotic chemotherapy, the main tool employed against clinical infections, is constantly threatened by increasing resistance in some organisms [2]. This necessitates a constant search for new antibacterials to preempt the resistance onslaught. Plants remain an important source of diverse chemical entities which have been used as drugs or provide scaffolds from which new drugs have been derived [3,4]. Consequently, the screening of plants for their medicinal value remains an active area of scientific investigation. The present paper describes some of the results of our attempt to discover new antibacterial drug leads from plant sources.

Antibacterial activity was demonstrated in the crude methanol extracts of Peperomia vulcanica and Peperomia fernandopoioana both from the Piperaceae family [5], and Scleria striatinux De Wild (Cyperaceae) [6]. These findings motivated our search for antibacterials from these plants. Twenty-nine pure compounds were isolated, seven of which were identified as: Okundoperoxide (1 or OKP), matairesinol dimethyl ether (2), 5-demethyltangeretin (3), stigmasterol (4), bursehernin (5), hexadecanoic acid (6) and linoleic acid (7). The antibacterial activity of all the plant secondary metabolites was evaluated and toxicity tests performed on the metabolite which was active.

## Materials and methods

### Plant materials

#### Collection and identification

Whole plants of P. vulcanica, Baker and C.H. Wright and P. fernandopoioana C.D.C. were collected from Mount Cameroon and authenticated by Mr. Ndive Elias, a botanist in the Limbe Biodiversity and Conservation Centre (LBCC), Cameroon. Voucher specimens N^O^ S.C.A. 8892 for P. vulcanica and N^O^ S.C.A. 8786 for P. fernandopoioana are available at the herbarium of LBCC.

The roots of Scleria striatinux De Wild (Cyperaceae) were collected in the North West Region of Cameroon, identified and assigned voucher specimen N^O^ 32235/HNC at the National Herbarium, Yaounde, Cameroon.

#### Preparation of crude extracts

**Figure 1:**
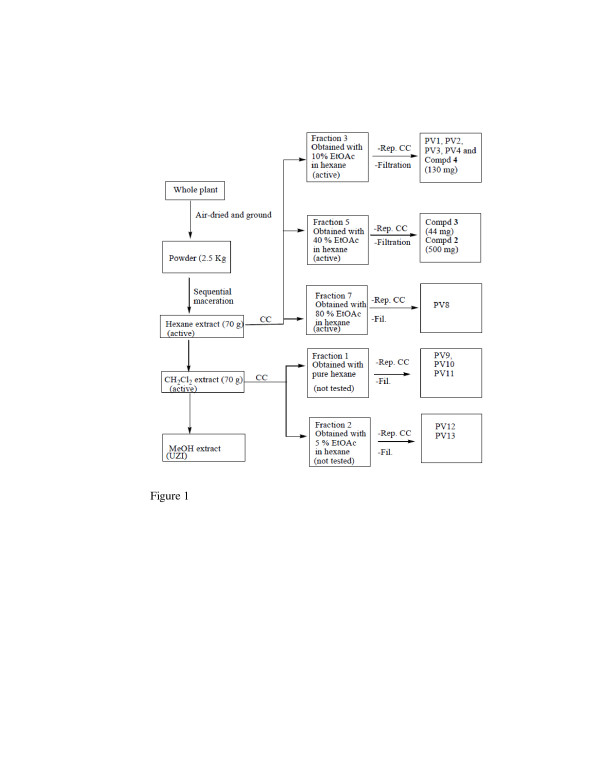
**Flow chart for the bioassay-guided fractionation and isolation of compounds from P.vulcanica.** PVs represent uncharacterized compounds. The identified compounds are numbered as in the text. UZI = unclear zone of inhibition.

**Figure 2:**
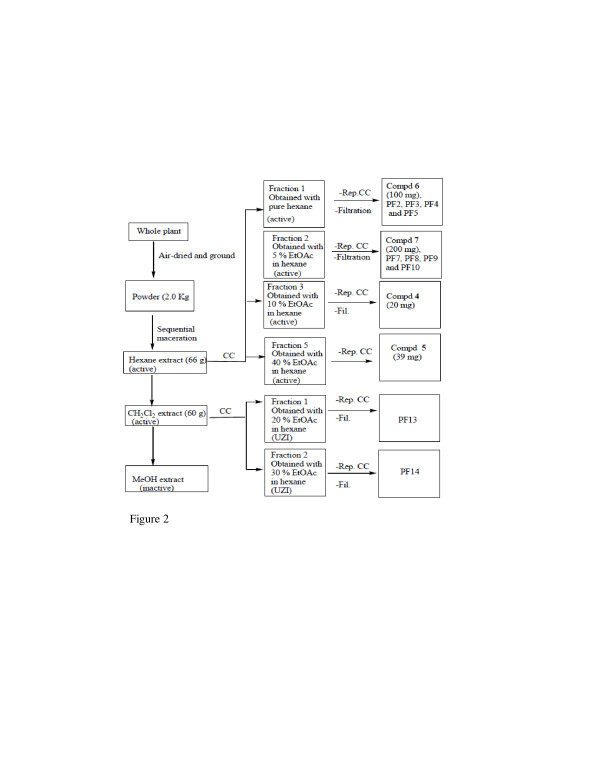
**Flow chart for the bioassay-guided fractionation and isolation of compounds from P. fernandopoioana.** PFs represent uncharacterized compounds. The identified compounds are numbered as in the text. UZI = unclear zone of inhibition.

Each plant material was air-dried at room temperature for one month, chopped and ground into powder which was further air-dried for 3 days. Each plant powder (2.5 g of P. vulcanica and 2 kg of P. fernandopoioana) was macerated for 48 hours three times per solvent and successively in hexane (6 L), methylene chloride (6 L) and methanol (partially done for biological testing). The mixture was filtered, and the filtrate concentrated by rotary evaporation. The concentrate was recovered with a small volume of methylene chloride and kept open at room temperature until all the residual solvent had evaporated. The dried crude extracts were weighed, and the bottles were sealed with Parafilm and stored at 4°C. The fractionation sequences are shown in Figures 1 and 2. For Scleria striatinux extraction was done as reported [7].

#### Fractionation of extracts and isolation of pure compounds

Bioassay-guided fractionation was done only on active crude extracts to isolate the pure compounds for further antibacterial screening. The hexane crude extract of P. vulcanica was fixed on Celite and fractionated using vacuum liquid chromatography (VLC) on silica gel and eluted with a gradient of ethyl acetate (EtOAc [0–80%]) in hexane. Following thin layer chromatography (TLC), 8 main fractions were identified (three of which were active). Fraction 3 (obtained with 10% EtOAc-hexane) was further chromatographed on SiO_2_ with a gradient of EtOAc in hexane to afford stigmasterol (130 mg). Fraction 5 (obtained with 40% EtOAc-hexane) was chromatographed on SiO_2_ and later passed through Sephadex LH-20 to yield 5-demethlytangeretin (44 mg) and white crystals of matairesinol dimethyl ether (500 mg) as shown on Figure 1.

The hexane extract of P. fernandopoioana (66 g) was fixed on Celite; repeated separation on silica followed by purification on Sephadex LH-20 yielded hexadecanoic acid (55 mg), linoleic acid and bursehernin (39 mg) as shown on Figure 2. This purification of the hexane crude extracts of both plants afforded a total of 20 pure products (8 from P. vulcanica and 12 from P. fernandopoioana). Similarly, fractionation and purification of the methylene chloride crude extracts using the same methods afforded 5 compounds from P. vulcanica and 2 compounds from P. fernandopoioana, making a total of 20 and 7 compounds for these plants from the hexane and methylene chloride extracts respectively.

Two compounds, one named Okundoperoxide (1 or OKP) and the other yet to be identified, were isolated from the CH_2_Cl_2_/MeOH (1:1) extract of S. striatinux [7]. Characterization of compounds was done using spectroscopic techniques and comparison of ^1^ H and ^13^ C Nuclear Magnetic Resonance (NMR) data with those found in the literature.

#### Bacterial cells

Four bacterial species, i.e. Escherichia coli, Staphylococcus aureus, Salmonella typhimurium, and Pseudomonas aeruginosa, were isolated from pathological specimens obtained from Solidarity Clinic, Molyko, Buea Sub-Division, Cameroon, using selective media as described [8,9].

Identification of isolates was based on their cultural, Gram reaction, morphological and specific biochemical characteristics. A strain of S. aureus resistant to vancomycin, ampicillin and erythromycin was also isolated. E. coli (ATCC 11775) and S. aureus (ATCC 33862) from the American Type Culture Collection were used as controls. The organisms were stored at 4°C and sub-cultured weekly during the study period.

#### Preparation of discs of crude extracts, fractions and pure compounds

Extract-impregnated discs were prepared as earlier described [10] with some modifications. Briefly, 5 mm discs were punched from a stack of four Whatman filter papers and autoclaved. Ten (10) μL of test solution (containing 1 mg of crude extract or fraction in acetone) was transferred onto a disc and the latter was dried in an incubator at 55°C for 20 minutes. This step was repeated four times giving discs containing 5 mg of crude extract or fraction. Discs containing 50 to 500 μg of pure compound were prepared similarly. Commercial antibiotic discs of vancomycin (30 μg), erythromycin (15 μg), colistin (10 μg) and ampicillin (10 μg) were used as positive controls. A 5 mg/mL stock of gentamycin was diluted in distilled water and a control disc containing 1 μg prepared as described above. Negative control discs were also prepared using 50 μL of acetone added in 10 μL aliquots and dried as above.

### Antibacterial susceptibility test

#### Disc diffusion method

The disc diffusion method was used as previously described [5,10] with some modifications. Bacterial suspension (100 μL of 6 x 10^8^ CFUs/mL in 0.85% saline equivalent to McFarland 2) was uniformly spread on nutrient agar (Columbia agar base) in a culture plate. The test, negative and positive control discs, were aseptically fixed with gentle pressure on labeled positions on the bacterial spread. Only fractions prepared from crude extracts which initially showed activity in the disc test were tested. The plates were incubated for 18–24 hours at 37°C and the zones of inhibition measured.

#### Determination of minimum inhibitory concentration (MIC) and minimum bactericidal concentration (MBC)

This was done for the most active crude extracts i.e. active on at least two bacterial species, and the pure compound which was active following the disc diffusion test. A stock solution of crude extract (40 mg/mL) was prepared by completely dissolving 40 mg of plant extract in 200 μL of dimethyl sulfoxide (DMSO) and adding 800 μL of peptone water sugar medium [11]. A stock solution of the pure compound (4 mg/mL) was prepared similarly. The MIC was determined by microdilution in a 96-well microtitre plate in duplicate wells as earlier described [12] with some modifications.

Peptone water sugar (130 μL) was pipetted into each required well and 50 μL of extract solution added in the test wells. Positive and negative control wells contained 50 μL of 50 μg/mL gentamycin (12.5 μg/mL gentamycin final concentration) and 50μL of sterile 0.85% saline respectively. Bacterial suspension (20 μL of 6 × 10^5^ CFUs/mL) was pipetted into all wells. This gave final concentrations of 0.5 - 10 mg/mL of the extract with 5% DMSO in the highest concentration well. The plate was incubated at 37°C for 24 hours. The lowest concentration that showed no bacterial growth (no colour change) was recorded as the MIC. The MIC of the active pure compound was determined using triplicate wells similarly as for crude extracts at a final concentration range of 50–1000 μg/mL. An aliquot (10 μL) of the MIC wells that showed no colour change were used to inoculate the surface of solid nutrient agar and incubated at 37°C for 24 hours. The lowest concentration of the inoculated MIC wells which showed no bacterial growth was recorded as the MBC.

### Toxicity studies

#### Cytotoxicity of okundoperoxide

The assay was carried out as earlier described [13] with some modifications using monkey kidney epithelial cells (LLC-MK_2_ from ATCC - CCL-7). Different concentrations (0.0078 - 8 mg/mL) of Okundoperoxide, which demonstrated antibacterial activity, were prepared in sterile-filtered RPMI-1640 culture medium (SIGMA), containing 100 μg/mL gentamycin and 2% DMSO final concentration. Cells were cultured in a 96-well plate. Prior to testing, the monolayers were washed and the test done in triplicate wells. Medium (150 μL) was introduced into all required wells. Then 50 μL of each solution of the compound was pipetted into corresponding wells. This gave wells with final concentrations of 1.95- 2000 μg/mL of the compound. Positive and negative control wells contained 0.125 M sodium azide and culture medium respectively. The plate was incubated at 37°C and observed for cell death over a period of 6 days. Dead or deformed cells, usually dark and rounded in shape, were counted by light microscopy and the LC_50_ and LC_100_ values determined graphically.

#### Acute toxicity of active extracts in mice

The test was conducted as described [14] with some modification and following the World Health Organization guidelines for the evaluation of safety and efficacy of herbal medicines [15]. Equal numbers of male and female Balb/c mice (21.34 ± 1.47 g) about 3 months old were used. They had access to food and water but were deprived of food 15–18 hours prior to the administration of the extracts. The mice were divided into 3 groups of six. The most active extracts of P. vulcanica, i.e. the hexane (PV_HEX_) and methylene chloride (PV_MC_) extracts (840 mg/mL each in peptone water sugar), were administered to separate groups of mice twice (i.e. 0.5 mL × 2 within 4 hours) giving a dose of 40 g/kg body weight [equivalent to 10 × MIC (4 mg/mL)], using an oral gauge. The control group received an equal volume (1 mL) of medium. The mice were observed for skin changes, mobility, sensitivity to pain (pinch) and mortality for 7 days. The animals were weighed before and after the experiment. At the end of the experiment, all animals were fasted overnight and sacrificed by cranial dislocation.

#### Statistical analysis of results

The diameter of zones of inhibition of crude extracts is reported as mean ± standard deviation (Table 1). The zones of inhibition of the active compound (Okundoperoxide) were reported in terms of percentage efficacy relative to the zones of standard antibiotics. The student’s *t*-test [Paired Two Samples for Means (n < 30)], was used to compare the experimental and control groups of animals before and after administration of the extracts. The t-values were calculated using SPSS - 17.0 software. A P value < 0.05 was considered statistically significant.

**Table 1:** Antibacterial activity of crude extracts of P. vulcanica, P. fernandopoioanaand Scleria striatinux

	Zone of inhibition (mm diameter)Ψ							
Organisms	Positive control	PV_HEX_	PV_MC_	PV_MeOH_	PF_HEX_	PF_MC_	PF_MeOH_	SS
E. *coli* (ATCC 11775)	GEN	9 ± 1	11 ± 0	10* ± 0	9 ± 1	11 ± 1	_	11
	25 ± 1							
E. *coli* (Path)	GEN	10 ± 0	10 ± 1	9* ± 0	9 ± 1	11 ± 0	_	13
	24 ± 2							
S. aureus (ATCC 33862)	VAN	8 ± 1	7 ± 0	_	_	7 ± 1	_	10*
	18 ± 1							
S. aureus (Path)	GEN 27*,	_	9 ± 1	_	_	7 ± 0	_	10*
	15 ± 1**							
S. typhimurium (Path)	VAN	_	_	_	_	_	_	_
	19 ± 1							
P. aeruginosa (Path)	GEN	_	_	_	_	_	_	_
	26 ± 1							

Ψ: Zones of inhibition for negative control (acetone) discs were = 0 mm Values are mean ± standard deviation of duplicates for 5 mg extract/disc. PV_HEX_, PV_MC_, PV_MeOH_ = hexane, methylene chloride and methanol extracts of P. vulcanica respectively. PF_HEX,_ PF_MC_, PF_MeoH_ = hexane, methylene chloride and methanol extract of P. fernandopoioana respectively, SS = methylene chloride:methanol (1:1) extract of Scleria striatinux, GEN = Gentamycin (1 μg) and VAN = Vancomycin (30 μg). – = No zone of inhibition observed. * = The outer portion (12 mm) of the whole inhibition zone (27 mm) was unclear. ** = clear inner portion of the whole 27 mm inhibition zone. Path = clinical isolate.

## Results

### Identification of compounds

The hexane and methylene chloride extracts of P. vulcanica (Figure 1) and P. fernandopoioana (Figure 2) were subjected to bioassay-guided fractionation. The active fractions were systematically chromatographed on silica gel and/or Sephadex LH-20 leading to the isolation of 27 compounds.

**Figure 3:**
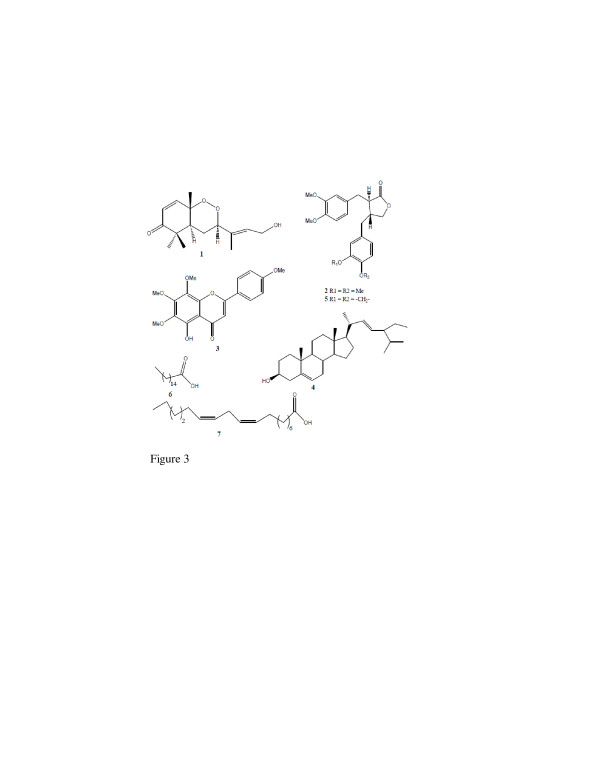
**Structures of seven of twenty-nine pure compounds isolated and screened for antibacterial activity.** Legend: Okundoperoxide (1) from S. striatinux, the only active compound was bacteriostatic on S. aureus; Matairesinol dimethyl ether (2), 5-Demethyl tangeretin (3), and Stigmasterol (4) from P. vulcanica; Bursehernin (5), Hexadecanoic acid (6) and Linoleic acid (7) from P. fernandopoioana.

Two compounds were obtained from Scleria striatinux. The structures of the isolated compounds (Figure 3) were determined by comparison of their spectral data with those reported for Okundoperoxide (1) [7], matairesinol dimethyl ether (2) [16], 5-demethyltangeretin(3) [17], stigmasterol (4) [18], bursehernin (5) [16], hexadecanoic acid (6) [19] and linoleic acid (7) [20].

### Antibacterial activity of test substances

#### Crude extracts

All seven plant crude extracts shown on Table 2 were screened against 6 bacterial test organisms. The diameter of the zones of inhibition (DZI) ranged from 7–13 mm (Table 1). The methylene chloride extracts of P. vulcanica, P. fernandopoioana and the methylene chloride:methanol (1:1) extract of S. striatinux (SS) were active against 4 of the 6 test organisms, i.e. the clinical and control strains of E. coli and S. aureus (Table 1). The hexane extract of P. vulcanica was active against three organisms while the methanol extract of P. vulcanica and the hexane extract of P. fernandopoioana were active against two organisms (Table 1). The methanol extract of P. vulcanica (PV_MeOH_) showed an unclear zone of inhibition (UZI, with visible colony forming units (CFUs) uniformly distributed in the inhibition zone) against the two E. coli test organisms (UZI ranging from 9–10 mm), while the methanol extract of P. fernandopoioana (PF_MeOH_) was inactive on all the test organisms. None of the extracts showed activity against the clinical isolates of S. typhimurium and P. aeruginosa (Table 1).

**Table 2:** Percentage yield (% w/w) of plant extracts

Plants	Dry weight of powder (Kg)	Extraction solvent			
		Hexane	Methylene	Methanol	MC: MeOH
		(HEX)	chloride (MC)	(MeOH)	(1:1)
		% yield	% yield	% yield	% yield
Peperomia vulcanica	1.75	3.58	3.78	N*	N
Peperomia fernandopoioana	2.0	3.3	2.97	N*	N
Scleria striatinux	8.5	N	N	N	11.76

N: not done. N*: partially done for preliminary testing.

#### Activity of fractions of active crude extracts

Each of the 8 hexane fractions of P. vulcanica showed activity against at least one of the 6 test organisms, and the zones of inhibition ranged from 6–11 mm. The methylene chloride fractions of P. vulcanica that were not tested in this study will be further investigated subsequently. The methanol crude extract was not fractionated due to the weak activity observed from the unclear zone of inhibition produced (Table 1). For P. fernandopoioana, a total of 8 hexane fractions and 7 methylene chloride fractions were screened. Only 4 hexane fractions showed clear zones of inhibition (DZI ranging 8–11 mm) against the two strains of S. aureus. Four other hexane and all seven methylene chloride fractions showed unclear zones of inhibition (UZI ranging from 7 - 11 mm) against S. aureus (ATCC 33862), and the two strains of E. coli. None of the fractions of P. vulcanica and P. fernandopoioana was active against the clinical isolates of S. typhimurium and P. aeruginosa. The methanol extract of P. fernandopoioana was not fractionated since it showed no activity.

#### Activity of pure compounds

A total of 29 pure compounds from the 3 plants were screened by the disc diffusion method at 50 to 500 μg/disc. None of the 13 compounds from P. vulcanica and the 14 from P. fernandopoioana showed activity against any of the test organisms at the highest amount (500 μg) tested. One of two compounds from Scleria stiatinux, Okundoperoxide, inhibited the two strains of S. aureus only. Two clinical isolates of S. aureus, one sensitive to five standard antibiotics and another resistant to three antibiotics with complete (100%) resistance to vancomycin (Table 3) identified during this study were susceptible to Okundoperoxide (OKP) at 500 μg/disc. OKP showed an efficacy ranging from 61.29 to 135.71% on the sensitive strains relative to five standard antibiotics; and an efficacy of 35.71 to 71.42% relative to four antibiotics on an isolate which was completely resistant to vancomycin i.e. DZI = 00 mm giving a relative efficacy of > > 100% for OKP compared to vancomycin (Table 3).

**Table 3:** Susceptibility of S. aureus to Okundoperoxide and standard antibiotics

Bacterial isolates	Zone of inhibition (mm) (% Efficacy of Okundoperoxide)					
	GEN	COL	ERY	AMP	VAN	OKP
S. *aureus* (path)	28	14	27	31	21	19
Sensitive strain	(67.86%)	(135.71%)	(70.37%)	(61.29%)	(90.48%)	
S. aureus	28*	14	24*	14*	00	10*
Resistant strain	(35.71%)	(71.42%)	(41.66)	(71.42%)	(> > 100%)	

GEN=Gentamycin (1 μg), COL=Colistin (10 μg), ERY=Erythromycin (15 μg), AMP=Ampicillin (10 μg), VAN=Vancomycin (30 μg), OKP = Okundoperoxide, isolated from S. striatinux (500 μg/disc) * = unclear zone of inhibition.

#### MICs and MBCs of active crude extracts and okundoperoxide

The MICs of active crude extracts and Okundoperoxide were determined for organisms on which the test substances were active. The lowest MIC for the crude extracts was 4 mg/mL and 1000 μg/mL for OKP (Table 4). The contents of the MIC wells for both the crude extracts and OKP which showed inhibition also showed bacterial growth on solid nutrient agar. Hence no MBC was recorded within the concentration ranges tested indicating that the active substances were only bacteriostatic.

**Table 4:** Minimum inhibitory concentrations (MICs) of active extracts and Okundoperoxide

Organisms	MIC (mg/mL)				
	PV_HEX_	PV_MC_	PF_MC_	SS	OKP
E. *coli* (ATCC 11775)	4	4	8	> 10	–
E. coli (Path)	4	4	8	> 10	–
S. aureus (ATCC 33862)	4	4	8	> 10	1
S. aureus (Path)	4	4	8	> 10	1

PV_HEX_, PV_MC_ = hexane and methylene chloride extracts of P. vulcanica respectively. PF_MC_ = methylene chloride extract of P. fernandopoioana. SS = methylene chloride:methanol (1:1) extract of Scleria striatinux. OKP = Okundoperoxide, isolated from S. striatinux. Path = clinical isolate (pathological), – = experiment not done

#### Cytotoxicity and acute toxicity

The cytotoxicity assay of Okundoperoxide on monkey kidney cells (LLC-MK_2_) recorded an LC_50_ =46.875 μg/mL and LC_100_ = 500 μg/mL. For the acute toxicity test, no death was recorded in both the control and test animals during the one week study period. However, the mice treated with the methylene chloride extract of P. vulcanica showed a group average weight loss of 13.21% which was statistically significant (P < 0.05) and had scanty fur. On the contrary, animals in the control group and those treated with the hexane extract of P. vulcanica gained weight (+5.01% and +3.54% respectively) but this was not significant; no other sign of toxicity was observed.

## Discussion

The Peperomias constitute a large genus of herbs widely distributed in tropical and sub-tropical regions [21]. The two species used in this study are found around Mount Cameroon in the South West and in parts of the North West of Cameroon [22]. Scleria striatinux is also found in the North West of Cameroon. Crude extracts of the three plants, P. vulcanica, P. fernandopoioana and S. striatinux investigated in this study all demonstrated antibacterial activity against some of the bacterial organisms except for the methanol extract of P. fernandopoioana. From the flow charts (Figures 1 and 2), one could observe clear activity in the fractions of moderate polarity. Both Gram-negative (E. coli, S. typhimurium and P. aeruginosa) and -positive bacteria (S. aureus) were selected based on their frequent occurrence in wounds and implication in common bacterial diseases such as diarrhoea, urinary tract infections and respiratory tract infections associated with coughing. Four clinical isolates were sensitive to standard antibiotics and one was a resistant strain of S. aureus that showed total resistance to vancomycin with a zero diameter of inhibition and intermediate resistance to erythromycin and ampicillin. Isolation of a resistant strain in the study area is not unexpected given the abusive use of antibiotics which favours the emergence of resistance as earlier described [23].

Six of the seven crude extracts from the three plants inhibited two of four bacterial species tested. The methylene chloride extracts of the Peperomia species and the methylene chloride:methanol (1:1) extract of Scleria striatinux were the most active (Table 1). Antibacterial activity had been demonstrated in the crude methanolic extracts of the whole plant of these two Peperomia species [5]. The earlier work constituted a basis for this study and our results confirm their findings and provide further evidence to support the use of these plants in Cameroonian folk medicine to treat bacterial infections [22]. The methanol extract of P. fernandopoioana showed no activity against all 6 bacterial test organisms, probably due to absence of compounds active against the bacteria following the sequential extraction performed on the plant powder.

The crude hexane extract of P. vulcanica showed no activity against the clinical isolate of S. aureus (Table 1). Interestingly, 3 of the 8 fractions obtained from the hexane extract showed moderate activity against this same sample with DZI = 10 mm suggesting antagonism between compounds in the crude extract which could have been eliminated following fractionation. These fractions obtained with moderately polar solvents were the most active with clear zones of inhibition; this suggests that the bio-active principles in these fractions are moderately polar. The hexane and methylene chloride extracts of P. fernandopoioana showed moderate activity against E. coli and S. aureus (Table 1). However, the activity of fractions from these extracts was not certain given the unclear zones observed. The latter observation suggests that the antibacterial action of the crude extracts of this plant may be due to the combined effect of the compounds present in them. Upon separation by fractionation, the antibacterial action was weakened or the active principles in the fractions were too small to be isolated in sufficiently high amounts to show activity.

Several plants have been reported to display antibacterial activity on the bacterial species used in this study [24-27]. The current study employed quantities of crude extract similar to those used in an earlier study and similar zones of inhibition were recorded, i.e. ranging from 7–15 mm as against 7–13 mm in this study. Also, much lower MICs were recorded (32–512 μg/mL against 4 - 8 mg/mL in this study). This higher activity is likely due to the secondary metabolites in the plant which was rich in alkaloids, flavonoids, phenol, quinines, and terpenoids [24]. Another study [25] also observed higher activity for six Phyllantus species having a similar phytochemical profile. The Peperomias tested in this study contained lignans, steroids, flavonoids and fatty acids. The difference in phytochemical composition may largely account for the difference in the reported activity while other factors such as experimental method may contribute to a smaller extent. This explanation is supported by yet similar findings on a plant with a similar secondary metabolite composition [26]. One of the investigations [27] reported much higher inhibition zones (14 - 36 mm) for the same bacteria species alongside higher MICs (4–64 mg/mL) and MBCs (8 - 128 mg/mL); these higher zones may be due to the high amount of extract (25 mg/disc) used.

Previous studies have shown that most of the common phytochemical constituents of Peperomia species are phenylpropanoid, benzopyran, chromone, prenylated quinone, secolignan, and acylcyclohexane-1, 3-dione [21,22]. Some of these compounds in the crude extracts and fractions of P. vulcanica and P. fernandopoioana may be responsible for the antibacterial activities observed.

The isolation of a large number of compounds (27 in total) from the two Peperomia species is not surprising. Nineteen compounds were isolated from P. sui of the same family [21] suggesting that the Piperaceae may be quite rich in secondary metabolites. Hence, sequential extraction and bioassay-guided fractionation were done to narrow down the number of metabolites and increase our chances of obtaining active compounds. The residual methanol extracts turned out to be weak or inactive, suggesting that sequential extraction actually localized the active compounds in the hexane and methylene chloride extracts (containing moderately polar compounds). However, none of the compounds from the Peperomias showed antibacterial activity, suggesting that the observed antibacterial activity for some of the crude extracts and fractions is likely due to the synergistic or additive interaction of some of these compounds; but this remains to be demonstrated experimentally.

One of the compounds, Okundoperoxide (OKP), from S. striatinux showed considerably high activity against a resistant and sensitive strain of S. aureus (Table 3). Considering the multi-drug resistance that has developed in S. aureus [2], a bacterium with potentially serious pathology in humans [28], this activity is therefore highly significant. This finding provides a strong basis for explorative structure-activity relationship studies which may yield new potent antibacterial lead(s) with enhanced activity. Okundoperoxide had been isolated, its structure determined (Figure 3) and antiplasmodial activity demonstrated, justifying the use of the roots of the plant as herbal tea for fevers in Cameroon [7]. Menthol, a terpenoid isolated from Mentha longifolia L. leaves showed much higher activity (25 mm zone and MIC of 15.6 μg/mL) against S. aureus [29]. A higher activity (MIC = 64 μg/mL) was also recorded against S. aureus for three plant-derived triterpenoids which showed a high degree of synergism with standard antibiotics [30]. The effect of OKP combined with standard antibiotics should also be investigated to discover useful combinations with enhanced antibacterial action to combat resistant bacteria.

The lack of susceptibility in P. aeruginosa and S. typhimurium to the crude extracts, fractions or pure compounds may be attributed to several factors including multi-drug efflux pumps common in P. aeruginosa and S. typhimurium and the low permeability of the bacterial envelopes [31]. Efflux pumps extrude the drug from the cell before they attain an adequate concentration at the site of action [32]. Some studies have reported no inhibition zone for 24 plant extracts against S. typhimurim [33] and menthol against P. aeruginosa [29]. Whereas no antibacterial activity was also reported for 40 plant extracts against S. typhimurium and S. aureus using the disc diffusion method, considerable inhibition of these organisms was recorded using the tube dilution method [5]. This suggests the tube dilution method may permit a more rapid accumulation and concentration of active principles than the diffusion method. The higher concentration of active secondary metabolites obtained with the tube dilution method would translate into more efficient inhibition of bacterial growth.

Based on some classifications of antibacterial activity using the disc method [14,34], the most active extracts [the methylene chloride extracts of P. vulcanica, P. fernandopoioana, and the methylene chloride:methanol (1:1) extract of S. striatinux] exhibited moderate activity (DZI between 11–16 mm). On the same basis, the pure compound (Okundoperoxide) showed high activity against S. aureus with a DZI ranging from 10–19 mm. Based on the MICs recorded for E. coli and S. aureus (4–8 mg/mL) and the apparently high MBC (> 10 mg/mL), these plants can be classified as possessing broad-spectrum bacteriostatic activity. Similarly Okundoperoxide with a high MIC of 1 mg/mL may only be bacteriostatic against S. aureus.

The LC_50_ of Okundoperoxide on monkey kidney cells (LLC-MK_2_) was 46.88 μg/mL whereas the active dose was 500 μg/disc i.e. about 10 times the LC_50._ This suggests OKP may be toxic to mammalian cells. However, use of different formulations, disc for antibacterial activity and solution for cytotoxicity makes comparison of the results difficult as the rate of distribution of the compound in both experimental systems is likely to be different. An LC_50_ = 10.02 μg/mL has been suggested to indicate moderate cytotoxicity [35], while a CC_50_ > 30 μg/mL has been categorized as non-cytotoxic [36]. Thus OKP with a higher LC_50_ can be considered as having a very low risk of cytotoxicity on mammalian cells. For P. vulcanica, no mortality was recorded in the acute toxicity study, suggesting that despite containing a large number of compounds this plant may be non-toxic to humans. The changes in body weight of the animals could have resulted from corresponding changes in feeding due to alteration of appetite or effects on the metabolism of the animals.

In conclusion, the observed antibacterial activity of the two Peperomia species is likely due to the combined effect of the moderately polar compounds present in them. Furthermore, the apparent lack of acute toxicity in P. vulcanica supports its use in traditional medicine. In view of its potency and relatively low cytotoxicty, Okundoperoxide may serve as a template for the development of new antibiotics. Moreover, given the structural similarity that is found among the secondary metabolites of a given plant, the isolation of other compounds from S. striatinux may result in the identification of other interesting antibacterial agents.

## Abbreviations

MIC: Minimum Inhibitory Concentration; MBC: Minimum Bactericidal Concentration; DZI: Diameter of Zones of Inhibition; LC50: Concentration which kills 50% of whole organism or cells; WHO: World Health Organization; LBCC: Limbe Biodiversity and Conservation Centre; HEX: Hexane; MC: Methylene chloride; MeOH: Methanol; EtOAc: Ethyl acetate; TLC: Thin Layer Chromatography; NMR: Nuclear magnetic resonance; ATCC: American Type Culture Collection; DMSO: Dimethyl sulfoxide; CFUs: Colony forming Units; LLC-MK2: Monkey kidney epithelial cells; RPMI-1640: Culture medium; SS: Scleria striatinux; UZI: Unclear Zone of Inhibition; OKP: Okundoperoxide; TWAS: Academy of Science for the Developing World; MMV: Medicine for Malaria Venture; CC50: Concentration which kills 50% of cells.

## Competing interests

The authors declare that they have no competing interests.

## Authors’ contributions

SMNE and JAM did the conception, design and supervision of the chemistry aspects of the work and contributed in writing the manuscript. KDN, SBB and LNN were involved in collection of plant materials and bench chemistry work. MNN designed and supervised the biological experiments and contributed in writing the manuscript. NDL contributed in the design, supervision of the bench work and drafting of the manuscript. ALA carried out the biological experiments. All authors read the manuscript, contributed in correcting it, and approved its final version.

## Authors’ information

SMNE is a Professor of Chemistry and Principal investigator with several research grants leading a team working on the medicinal chemistry of medicinal plants among other areas. JAM holds a PhD in Chemistry and is interested in drug discovery from medicinal plants. MNN holds a PhD in Biochemistry and works on discovery and development of antimicrobials. NDL holds a PhD in Biochemistry and works on protein chemistry and drug targets. KDN is a PhD student while SBB, LNN and ALA are MSc students; they were involved in this study as part of the thesis for their respective degrees.

## References

[B1] WHO (World Health Organization) Technical Health Report: bacterial challenge 2009 8 23

[B2] Zhang R Eggleston K Rotimi V Zeckhauser RJ Antibiotic resistance as a global threat: Evidence from China, Kuwait and the United States Global Health 2006 2 6 10.1186/1744-8603-2-6 16603071 PMC1502134

[B3] Cowan MM Plant Products as Antimicrobial Agents Clin Microbiol Rev 1999 12 564 582 10515903 10.1128/cmr.12.4.564 PMC88925

[B4] Raskin I Ribnicky DM Komarnytsky S Ilic N Poulev A Borisjuk N Brinker A Moreno DA Ripoll C Yakoby N O’neal JM Cornwell T Pastor I Fridlender B Plants and human health in the twenty - first century Trends Biotechnol 2002 20 522 531 10.1016/S0167-7799(02)02080-2 12443874

[B5] Ngemenya MN Mbah JA Tane P Titanji VPK Antibacterial effects of some Cameroonian medicinal plants against common pathogenic bacteria Afr J Trad CAM 2006 3 84 93

[B6] Ndip RN Tarkang AEM Mbullah SM Luma HN Malongue A Ndip LM Nyongbela K Wirmum C Efange SMN In vitro anti-Helicobacter pylori activity of extracts of selected medicinal plants from North West Cameroon J Ethnopharmacol 2007 114 452 457 10.1016/j.jep.2007.08.037 17913416

[B7] Efange SMN Brun R Wittlin S Connolly JD Hoye TR Akam TM Makolo F Mbah JA Nelson DP Nyongbela KD Wirmum C Okundoperoxide, a bicyclic cyclofarnesyl sesquiterpene endoperoxide from Scleria striatimux with antiplasmodial activity J Nat Prod 2009 72 280 283 10.1021/np800338p 19199815 PMC2765531

[B8] Ndip RN Dilonga HM Ndip LM Akoachere JFK Akenji TN Pseudomonas aeruginosa isolates recovered from clinical and environmental samples in Buea, Cameroon: current status on biotyping and antibiogram Trop Med Int Health 2005 1 74 81 15655016 10.1111/j.1365-3156.2004.01353.x

[B9] Cheesbrough M Antimicrobial sensitivity testing District Laboratory practice in tropical countries Part II 2000 Cambridge University Press, Cambridge 132 143

[B10] Cheesbrough M Medical laboratory manual for Tropical Countries Part II 1984 Butterworth- Heinemann, Oxford, UK 196 200

[B11] Cheesbrough M District Laboratory Practice in Tropical Countries Part II 2000 Cambridge University Press, 401 402

[B12] Sarker SD Nahar L Kumarasamy Y Microtitre plate-base antibacterial assay incorporating resazurin as an indicator of cell growth, and its application in the in vitro antibacterial screening of phytochemicals Science 2007 42 321 324 10.1016/j.ymeth.2007.01.006 PMC1895922 17560319

[B13] Leon-Díaz R Meches M Fernandez SS Salinas GMM Villarreal JV Torres J Harrera JL Arellanes AJ Antimycobacterial neolignans isolated from Aristolochia taliscana Mem Inst Oswaldo Cruz 2010 105 45 51 10.1590/S0074-02762010000100006 20209328

[B14] Assam AJP Dzoyem JP Pieme CA Penlap VB In vitro antibacterial activity and acute toxicity studies of aqueous-methanol extract of Sida rhombifolia Linn. (Malvaceae) BMC Complement Altern Med 2010 10 40 10.1186/1472-6882-10-40 PMC2922083 20663208

[B15] WHO (World Health Organization) Research guidelines for evaluating the safety and efficacy of herbal medicines 2010

[B16] Estevez-Braun A Estevez-Reyes R Gonzalez A 13 C NMR assignments of some dibenzyl-c-butyrolactone lignans Phytochemistry 1996 43 885 886 10.1016/0031-9422(96)00363-9

[B17] Dugo P Bonaccorsi I Ragonese C Russo M Donato P Santi L Mondello L Analytical characterization of mandarin (Citru deliciosa Ten) essential oil Flavour Fragr J 2010 26 34 46

[B18] Holland LH Diakow RPP Taylor JG 13 C nuclear magnetic resonance spectra of some C-19 hydroxy, C-5,6 epoxy, C-24 ethyl and C-19-norsteroids Can J Chem 1978 56 3121 3126 10.1139/v78-510

[B19] Neol BK Rahayu UU Nordin HL Tai YC Tu YL Mohd AS Chemical constituents from two weed species of Spermacoce (Rubiaceae) Malaysian J Anal Sci 2010 14 6 11

[B20] Ashraf T Fang-Rong C Yue-Han L Yuh-Chwen C Chil-Chuang L Patnam R Chemical constituents from Hydrangea chinensis Arch Pharm Res 2003 26 15 20 10.1007/BF03179924 12568351

[B21] Cheng MJ Chen IS Secondary metabolites from Peperomia sui J Chil Chem Soc 2008 53 1539 1542

[B22] Mbah JA Tchuendem MHK Tane P Sterner O Two chromones from Peperomia vulcanica Phytochemistry 2002 60 799 801 10.1016/S0031-9422(02)00191-7 12150802

[B23] Akoachere TK Ndip RN Chenwi EB Ndip LM Njock TE Anong DN Antibacterial effect of Zingiber officinale and Garcinia kola on respiratory tract pathogens East Afr Med J 2002 79 588 592 12630492 10.4314/eamj.v79i11.8804

[B24] hameethunisa Hopper W Antibacterial activity of Artemisia nilagirica leaf extracts against clinical and phytopathogenic bacteria BMC Complement Altern Med 2010 10 6 http://www.biomedcentral.com/1472-6882/10/6 10.1186/1472-6882-10-6 20109237 PMC2830175

[B25] Komuraiah A Bolla K Rao KN Ragan A Raju VS Singara Charya MA Antibacterial studies and phytochemical constituents of South Indian Phyllanthus species Afr J Biotechnol 2009 8 4991 4995 http://www.academicjournals.org/AJB

[B26] Hidayathulla S Chandra KK Chandrashekar KR Phytochemical evaluation and antibacterial activity of Pterospermum diversifolium Blume Int J Pharm Pharm Sci 2011 3 165 167

[B27] Obeidat M Shatnawi M Al-alawi M Al-Zu’bi E Al-Dmoor H Al-Qudah M El-Qudah J Otri I Antimicrobial activity of crude extracts of some plant leaves Res J Microbial 2012 7 59 67 10.3923/jm.2012.59.67

[B28] Kumar P Clark M Saunders WB Infectious diseases, tropical medicine and sexually transmitted diseases Clinical Medicine 2002 5 Elsevier Science Limited, Edungburgh 27

[B29] Al-Bayati FA Isolation and identification of antimicrobial compound from Mentha longifolia L. leaves grown wild in Iraq Ann Clin Microbiol Antimicrob 2009 8 10.1186/1476-0711-8-20 PMC2707363 19523224

[B30] Chung PY Navaratnam P Chung LY Synergistic antimicrobial activity between pentacyclic triterpenoids and antibiotics against Staphylococcus aureus strains Ann Clin Microbiol Antimicrob 2011 10 25 10.1186/1476-0711-10-25 21658242 PMC3127748

[B31] Cornelis P Pseudomonas: Genomics and Molecular Biology 2008 1 Caister Academic Press, Norwich, England

[B32] Tenover FC Mechanisms of Antimicrobial Resistance in Bacteria Am J Med 2006 119 Suppl 6A S3 S10 16735149 10.1016/j.amjmed.2006.03.011

[B33] Parekh J Chanda S Antibacterial and phytochemical studies on twelve species of Indian medicinal plants Afr J Biomed Res 2007 10 175 181

[B34] Monks R Noel CL Amelia HT Farias FM Elfrides SES Suyenga SS Da Rocha AB Schwartsmann G Mothes B Anticancer, antichemotactic and antimicrobial activities of marine sponges collected off the coast of Santa Catarina, Southern Brazil J Exp Mar Biol Ecol 2002 281 1 12 10.1016/S0022-0981(02)00380-5

[B35] Khan A Rahman M Islam MS Antibacterial, antifungal and cytotoxic activities of 3,5- diacetyltambulin isolated from Amorphophallus campanulatus Blume ex. Decne DARU 2008 16 239 244 10.4103/0253-7613.40489 PMC3023122 21264161

[B36] Malebo HM Tanja W Caletal M Antiplasmodial, anti-trypanosomal, anti-leishmanial and cytotoxicity activity of selected Tanzanian medicinal plants Tanzan J Health Res 2009 11 226 234 20734703 10.4314/thrb.v11i4.50194

